# Epidemiological survey of human echinococcosis in east Gansu, China

**DOI:** 10.1038/s41598-021-85843-w

**Published:** 2021-03-18

**Authors:** Shuai Yan, Dong Wang, Junrui Zhang, Xiaojin Mo, Yu Feng, Liling Duan, Deyu Liu, Fan Li, Yongchun Dao, Ting Zhang, Wei Hu, Zheng Feng, Bin Zheng

**Affiliations:** 1 National Institute of Parasitic Diseases, Chinese Center for Disease Control and Prevention, WHO Collaborating Center for Tropical Diseases, National Center for International Research on Tropical Diseases, Key Laboratory of Parasite and Vector Biology of the Chinese Ministry of Health, Joint Research Laboratory of Genetics and Ecology on Parasite-Host Interaction, Chinese Center for Disease Control and Prevention & Fudan University, Shanghai, 200025 China; 2 Institute of Parasitic Diseases, Gansu Province Center for Disease Control and Prevention, Lanzhou, 730020 China; 3 The Endemic Disease Prevention Office in Huan County, Qingyang, 745700 Gansu China; 4 Department of Microbiology and Microbial Engineering, School of Life Sciences, Fudan University, Shanghai, 200438 China

**Keywords:** Parasitic infection, Epidemiology

## Abstract

Echinococcosis is endemic in pastoral regions of south, west and mid-Gansu province, China. The present study aimed to determine the prevalence of echinococcosis in east Gansu, and analyze its associated risk factors. A cross-sectional survey was conducted in 2011 in 12 villages of Xiaonangou township, Huan County in east Gansu province by ultrasound abdominal scan and auxiliary serotest, and a prevalence surveillance study from 2008 to 2014 was performed in one villages by ultrasonography screening. Questionnaire information analysis indicates that the risk factors are in association with the gender, age, and education level. The cross-sectional survey found a cystic echinococcosis prevalence of 2.21% (107/4837). Higher prevalence was seen in females (*χ*^2^ = 4.198, *P* < 0.05), older ages (> 60 years) (*χ*^2^_trend_ = 96.30, *P* < 0.05), and illiterates (*χ*^2^ = 90.101, *P* < 0.05). Prevalence surveillance showed changing profile of 3.35% in 2011 to 0.88% (1/113) in 2014.

## Introduction

Echinococcosis is a serious and potentially fatal zoonotic helminthic disease worldwide distributed, caused by larval stage of the genus *Echinococcus*^1,2^. Echinococcosis not only imposes a substantial health burden on families but also contributes to overburden the healthcare systems, and impedes socio-economic development. There are two main types of the disease: cystic echinococcosis (CE) caused by *Echinococcus granulosus* and alveolar echinococcosis (AE) caused by *E. multilocularis*^3–5^. CE has a cosmopolitan distribution and represents a major public health problem in some regions^6,7^. It is considered endemic in areas such as Peru, Chile, Argentina, Uruguay, southern Brazil, the Mediterranean region, central Asia, western China, and East Africa^8^. The life cycle of *E. granulosus* occurs in a synanthropic cycle through domestic dogs as definitive hosts and a variety of livestock species as intermediate hosts^9^. Humans can serve as an aberrant intermediate host, and become accidentally infected through ingestion of *E. granulosus* eggs directly via hand-to-mouth transfer after handling the infected definitive host or indirectly after consuming contaminated food or water^10^.

Human AE cases in Gansu were first identified in 1981^11^. The reported AE cases occurred in Zhang and Min Counties of south Gansu province, with the prevalence rate (3%) at a relatively stable level^10,12–14^. Noticeably, CE are reported from Minle and Tianzhu Counties of west Gansu province, and the recorded prevalence rates were in the range of 0.75–1.3%^13,15–17^. The current study site, Huan county, is located in the east of Gansu province, and a farming-pastrol region, where herding livestock, and keeping domestic dogs for guarding households and livestock are very common. In this area, *Echinococcus* infection in humans and animals was noted by local clinics^18^. However, little information is known on the echinococcosis status and transmission in east Gansu, due to lack of systematic population-based survey. Therefore, to estimate the echinococcosis prevalence, transmission trend and analyze the risk factors in east Gansu area, we conducted the current epidemiological survey comprising a cross-sectional community survey in 12 villages of Xiaonangou township, Huan county in 2011, a prevalence surveillance study in one of the villages from 2008 to 2014.

## Methods

### Study location

**Figure 1:**
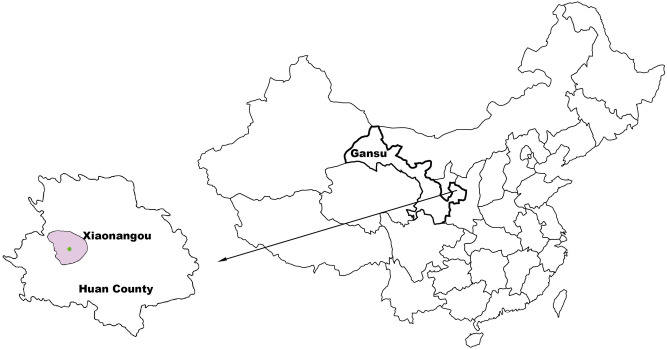
Map of the study location in Huan County, Gansu province of China. The green spot indicates the survey site Xiaonangou township. The map was generated in-house (the base map is available online http://d-maps.com) and was modified using Adobe software Illustrator C5.

**Figure 2:**
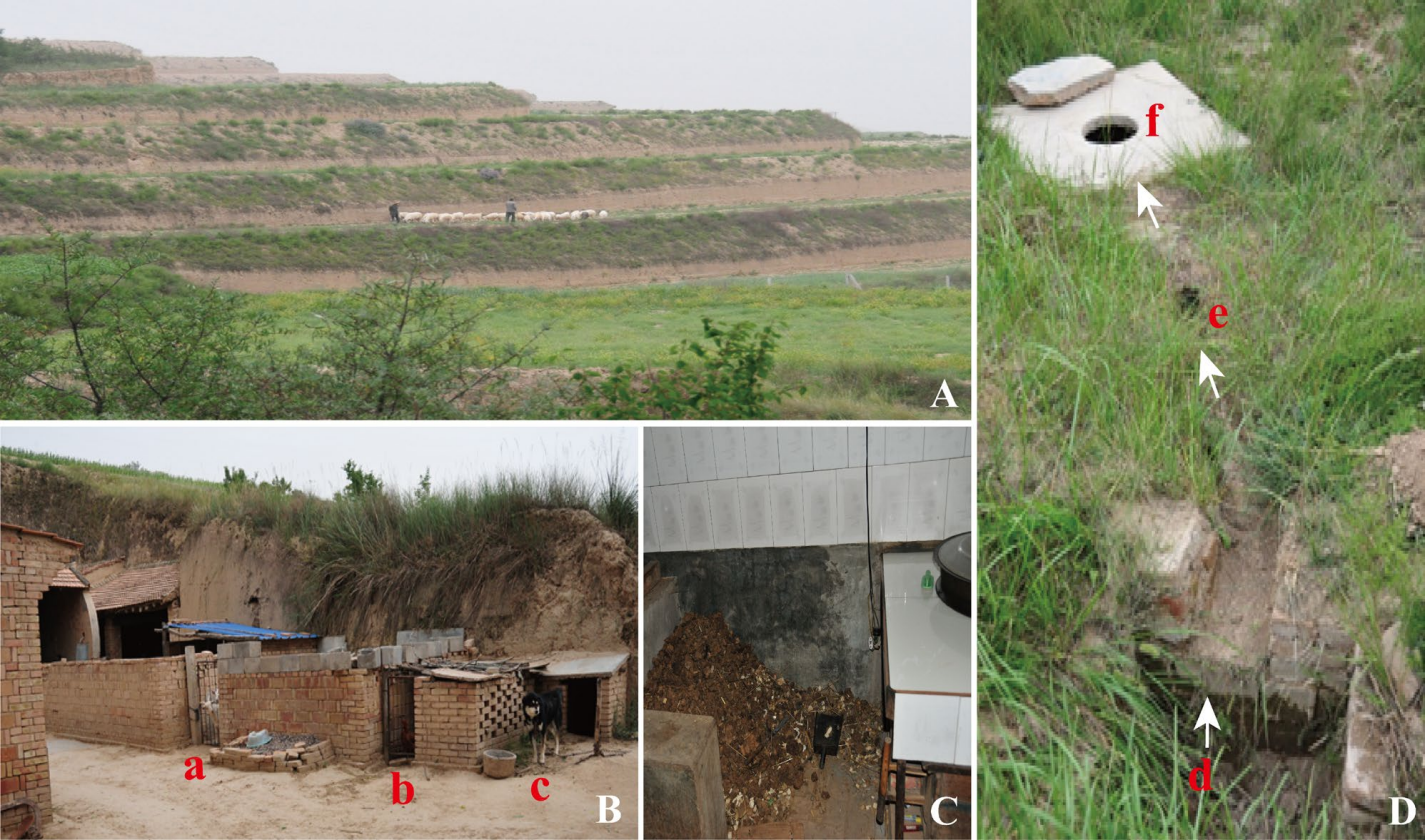
Households and transmission environment of echinococcosis in Xiaonangou township. (**A**) Grazing sheep in grassland, (**B**) Stalls rearing domestic animals adjacent to the house for (a) sheep, (b) chicken and (c) dog, (**C**) Cow dung stored in corner of kitchen as fuel supply for cooking, (**D**) Water cellar for collection of rainfall as drinking water for farmers and animals (d) inlet of rainfall collection pool for sedimentation, (e) water flow channel, (f) an opening of reservoir well for water uptake. The arrows indicate the water flow direction downwards from a higher position (d) through (e) to reservoir well (f) when the water overfills the collection pool.

The present study was conducted in Xiaonangou Township of Huan County, east Gansu province, a region located in central-western China, 36°37′ north latitude, and 106°49′ east (Fig. 1). The average altitude of the township is about 1920 m, covering an area of 582 km^2^ with a resident population of approximately 13,239, among them there are 1000 primary school children, and the local farmers owned approximately 2500 dogs. The majority of the residents engage in farming-pastoral production activity, which is the economic pillar of this region, thus almost every family raises sheep, cattle, chickens, and dogs. Furthermore, enclosed stalls for dogs, cattle, and sheep are set just next to farmer’s house. Traditionally, dried cow dung is used as a fuel source for cooking, which is piled in the kitchen (Fig. 2A–C). In addition, the main source of drinking water for residents and animals is the rainfall collected in traditional water cellars, which are built as a rain fall collection setting comprised of a well, drainage channel and an underground sedimentation pool (Fig. 2D). Apparently, the local environment providing chances for direct or indirect contact with animals and the water contaminated with parasite eggs may favor the transmission of echinococcosis.

### Study design, diagnosis and treatment

This study was sponsored by the Gansu Province Center for Disease Control, comprising two study parts: (1) a cross-sectional survey in 12 villages of Xiaonangou township in 2011, (2) a prevalence surveillance study in one village of the township from 2008 to 2014. All participants in the studies were volunteers self-selected from the study sites. The study purpose and procedure were explained to the community through village leaders. For risk factor analysis, a questionnaire investigation was carried out for all participants to collect demographic information including the residence address, gender, age, and education.

Specifically, the two studies were conducted as the following: (1) a cross-sectional survey enrolled a total of 4837 residents self-selected from 12 villages of Xiaonangou township in 2011; (2) a prevalence surveillance in Yanghutaozi village of Xiaonangou township with a total of 5121 residents participated were screened by ultrasonography in 2008–2014.

The participants in the cross-sectional survey were examined by abdominal ultrasound (US) scan. CE and AE were classified in accordance with the Diagnostic Criteria for Echinococcosis China (WS 257–2006), which is in line with the diagnostic standard developed by WHO-IWGE^19,20^. The diagnosis was achieved based on US pathognomonic image and confirmatory serological test ELISA. For those with dubious US images of space-occupying lesions, serum samples were collected for ELISA to detect specific IgG antibody against *Echinococcus* for confirming clinical infection. The US scan was performed by trained local health professionals using a portable ultrasound scanner (Mindray-DP3200) and a 2.0–12.0 MHz transducer. The ELISA was performed using a commercial ELISA kit (Haitai Biological Pharmaceuticals Co., Ltd., Zhuhai, China), with the protocol and reagents provided in the kit^21^. Briefly, the microtiter plate pre-coated with hydatid cyst fluid (HSF) antigen was used for applying serum samples. For colorimetric detection, horseradish peroxidase-conjugated mouse anti-human IgG antibody and an enzyme substrate were used sequentially by incubations at 37 °C. The resultant optical density was measured at 450 nm using a microplate reader (BioTek, USA). According to the cutoff OD value that the kit system defined, the sample was determined as positive or negative reaction. The sensitivity of the assay is 91%, and the specificity is 94.7%.

All the diagnosed echinococcosis cases, except pregnant women and children under age of 2 years, were treated with albendazole at a dosage of daily 10–15 mg/kg according to the WHO recommendations^22^. Surgical intervention was available at the hospitals in Lanzhou City, the capital of the province. When indicated, the local health care workers will arrange the surgical treatment.

### Data analysis

Microsoft Excel, SPSS software (version 19.0; SPSS Inc., Chicago, IL) and GraphPad Prism® software (San Diego, CA, USA) were used to compare the prevalence and analyze the risk factors in association with the demographic information. Basic descriptive analyses were performed, prevalence distribution and surveillance dynamics were analyzed using *Chi*-square (and Fisher’s exact) test, and *Chi*-square test for trend. *P* < 0.05 was examined statistically significant. The 95% confidence intervals were calculated for the sample size np ≥ 5 and n(1-p) ≥ 5, using the Normal Approx method; whereas for np < 5 or n(1-p) < 5, using Clopper-Pearson method.

### Ethics approval and participant consent

This study protocol was reviewed and approved by the Ethics Committee of Gansu Province Center for Disease Control and Prevention (No. 2018-001). All procedures in this study complied with the guidelines of the Ethics Committee, which is in line with the Declaration of Helsinki. Before the field work commencing, the study purpose and procedures were explained to the participants and/or guardians, and their written informed consent was obtained. All participants and guardians were provided examination and treatment when diagnosed of no costs; and could leave the program at any time; all personal data were kept confidential.

## Results

### Prevalence of echinococcosis in Xiaonangou township in 2011

In the cross-sectional survey, a total of 4837 participants selected from 12 villages in Xiaonangou township was examined, among them 107 (2.21%) individuals were diagnosed with CE. No AE case was found in the survey by US. Of the 107 diagnosed cases, 81 were US pathognomonic image positive, and 26 were confirmed of *Echinococcus* antibody positive by ELISA from 96 US dubious cases. Table 1 showed the prevalence in the 12 villages surveyed ranging between 1.37 and 6.80%, among them, higher prevalence was seen in three villages: Dingzhaike (4.18%), Fentaicha (6.80%), Yanghutaozi (3.35%), while 1–2% prevalence in other villages.

**Table 1:** Prevalence of cystic echinococcosis in 12 villages of Xiaonangou Township in 2011.

Villages	No. examined	No. cases	Prevalence % [95% CI]
Dingzhaike	335	14	4.18 [2.04–6.32]
Fenzishan	551	12	2.18 [0.96–3.40]
Fentaicha	147	10	6.80 [2.73–10.87]
Lishangshan	627	13	2.07 [0.96–3.19]
Liyuan	193	5	2.59 [0.35–4.83]
Lianchuan	534	8	1.50 [0.47–2.53]
Tianziqu	425	7	1.65 [0.44–2.86]
Wangtianzi	436	6	1.38 [0.28–2.47]
Xuzhang	219	3	1.37 [0.28–3.95]
Xiaonangou	855	16	1.87 [0.96–2.78]
Yanmaizhang	306	6	1.96 [0.41–3.51]
Yanghutaozi	209	7	3.35 [0.91–5.79]
Total	4837	107	2.21 [1.80–2.63]

CI: confidence interval.

**Figure 3:**
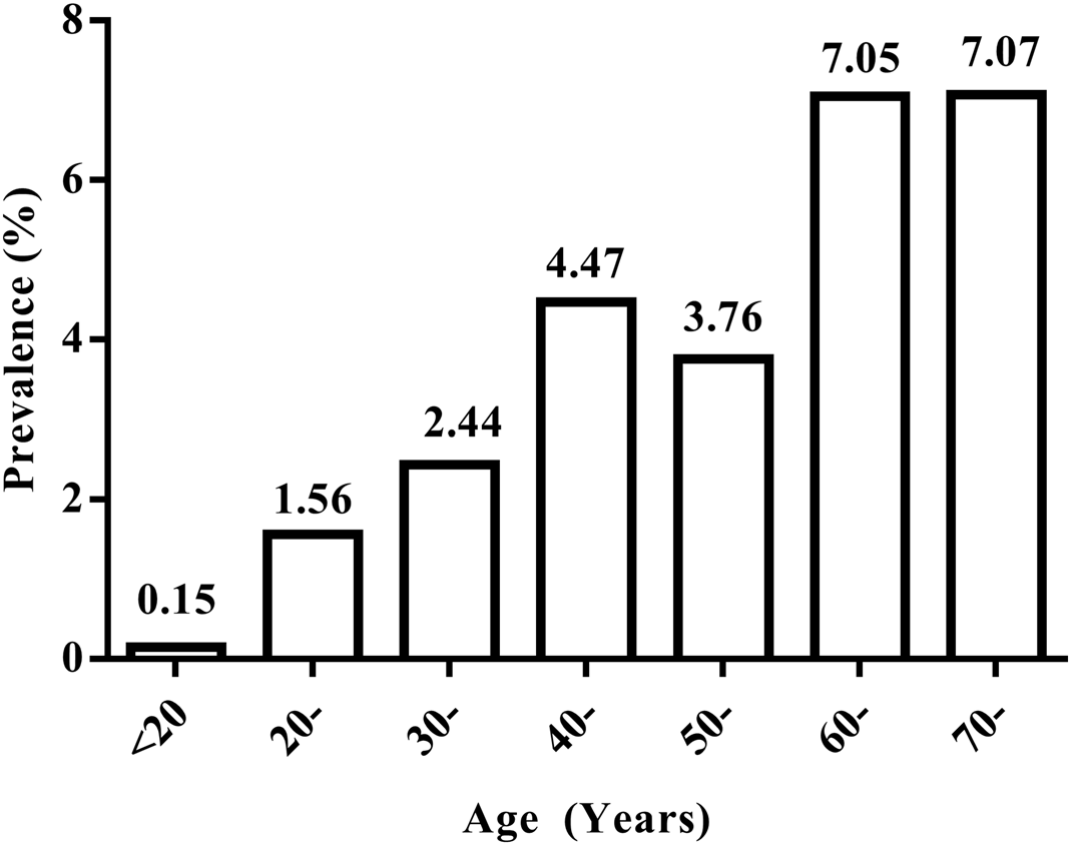
Prevalence of echinococcosis by age distribution in Xiaonangou township in 2011.

The age distribution of prevalence (age < 20, 20-, 30-, 40-, 50-, 60-, > 70) was illustrated in Fig. 3. Chi-square test for trend revealed that the prevalence increased with age (*χ*^2^_trend_ = 96.30, *P* < 0.05). The lowest prevalence (0.15%) was found in the age group of < 20, and then gradually increased, reaching a peak at the age over 60 (Fig. 3). Of total 4837 participants examined, 2708 were males, and 2129 females. The prevalence by gender distribution showed that the prevalence rate in females (2.72%, 58/2129) was significantly higher than that in males (1.81%, 49/2708) (*χ*^2^ = 4.198, *P* < 0.05). Compared by education level, the prevalences between the groups were significantly different (*χ*^2^ = 90.101, *P* < 0.05), with the highest prevalence in the illiterate group (7.05%, 47/667), and the lowest in the group of high school and higher (0.65%, 1/154) (Table 2).

**Table 2:** Prevalence of cystic echinococcosis by education level in Xiaonangou township in 2011.

Education	No. participants	No. cases	Prevalence % [95% CI]
Illiterate	667	47	7.05 [5.10–8.99]
Elementary school	2219	44	1.98 [1.40–2.56]
Middle school	1797	15	0.83 [0.41–1.26]
High school or higher	154	1	0.65 [0.02–3.57]
*χ*^2^-value			90.101
*P*-value			< 0.0001

CI: confidence interval.

### Prevalence surveillance in Yanghutaozi village from 2008 to 2014

The prevalence surveillance was conducted in Yanghutaozi village of Xiaonangou township from 2008 to 2014 in a total of 5121 community residents, among them 118 were diagnosed with CE by US screening (Table 3). During the surveillance years, no AE pathognomonic image was found, indicative of a CE endemic area, unlikely a CE-AE co-endemic area. It was noted that the prevalence was fluctuated in a range of 2–3% in 2008–2013, with the lowest 0.88% (11/996) in 2014 (Table 3).

**Table 3:** Prevalence surveillance of echinococcosis in Yanghutaozi village of Xiaonangou Township by ultrasonography screening in 2008–2014.

Year	No. examined	No. cases	Prevalence % [95% CI]
2008	2316	52	2.24 [1.64–2.85]
2009	1831	42	2.29 [1.61–2.98]
2010	228	6	2.63 [0.55–4.71]
2011	209	7	3.35 [0.91–5.78]
2012	303	9	2.97 [1.06–4.88]
2013	121	3	2.48 [0.51–7.08]
2014	113	1	0.88 [0.02–4.83]

CI: confidence interval.

The risk factor analysis showed that the prevalence in females was significantly higher than in males in 2008 and 2009 (*χ*^2^ = 6.368, 13.898, *P* < 0.05); however, no significant difference was observed in the other years (*P* > 0.05). The age distribution was analysed by Chi-square test for trend, showing that the prevalence increased significantly with age (*χ*^2^_trend_ = 124.4–5.37, *P* < 0.05) in 2008–2013. The education distribution indicates that the prevalences in different education groups were significantly different in 2008, 2009, 2012 and 2013 (*χ*^2^ = 61.825–10.248, *P* < 0.05), with the highest prevalence in the illiterate group seen in those years (Table 4).

**Table 4:** Risk factor analysis of cystic echinococcosis in Yanghutaozi village in 2008–2014.

Prevalence % [95% CI]							
	2008	2009	2010	2011	2012	2013	2014
**Gender**							
Male	1.54 (20/1295)	1.05 (10/956)	2.80 (4/143)	3.09 (3/97)	2.70 (4/148)	0.99 (1/101)	0 (0/64)
	[0.87–2.22]	[0.40–1.69]	[0.77–7.01]	[0.64–8.77]	[0.64–8.77]	[0.02–5.39]	–
Female	3.13 (32/1032)	3.66 (32/875)	2.35 (2/85)	3.57 (4/112)	3.23 (5/155)	10 (2/20)	2.04 (1/49)
	[2.04–4.16]	[2.41–4.90]	[0.29–8.24]	[0.98–8.89]	[0.44–6.01]	[1.24–31.70]	[0.05–10.85]
*χ*^2^-value	6.368	13.898	0	0	0	2.498	–
*P*-value	0.012	0.0002	> 0.9999	> 0.9999	> 0.9999	0.114	0.4336
**Age**							
< 20	0.09 (1/1121)	0.19 (1/514)	0.90 (1/111)	0 (0/66)	0 (0/72)	0 (0/23)	0 (0/15)
	[0.002–0.50]	[0.01–1.08]	[0.02–4.92]	–	–	–	–
20-	0 (0/288)	0.28 (1/353)	0 (0/24)	0 (0/17)	0 (0/21)	0 (0/11)	0 (0/11)
	–	[0.01–1.57]	–	–	–	–	–
30-	2.36 (9/381)	1.07 (3/281)	8.33 (2/24)	0 (0/24)	0 (0/47)	3.33 (1/30)	0 (0/13)
	[0.84–3.89]	[0.22–3.09]	[1.03–27.00]	–	–	[2.11–26.53]	–
40-	5.04 (17/337)	3.43 (11/321)	2.08 (1/48)	9.52 (4/42)	1.22 (1/82)	0 (0/41)	0 (0/15)
	[2.71–7.38]	[1.44–5.42]	[0.05–11.07]	[0.66–22.62]	[0.03–6.61]	–	–
50-	11.2 (14/125)	8.24 (14/170)	5.26 (1/19)	3.85 (1/26)	5 (2/40)	0 (0/14)	4.55 (1/22)
	[5.67–16.73]	[4.10–12.37]	[0.13–26.03]	[0.10–19.64]	[0.61–16.92]	–	[0.11–22.84]
60-	18.18 (10/55)	6.76 (10/148)	100 (1/1)	0 (0/11)	18.18 (6/33)	100 (2/2)	0 (0/33)
	[7.99–28.37]	[2.71–10.80]	–	–	[5.02–31.34]	–	–
> 70	11.11 (1/9)	4.55 (2/44)	0 (0/1)	8.7 (2/23)	0 (0/8)	0 (0/0)	0 (0/4)
	[0.28–48.25]	[0.56–15.47]	–	[1.07–28.04]	–	–	–
*χ*^2^_trend_-value	124.4	47.74	4.89	4.82	13.87	5.37	0.2
*P*-value	< 0.0001	< 0.0001	0.027	0.028	0.0002	0.02	0.65
**Education**							
Illiteracy	5.93 (40/675)	5.05 (22/436)	7.14 (2/28)	4.17 (1/24)	8.57 (6/70)	33.33 (2/6)	0 (0/25)
	[4.15–7.71]	[2.99–7.10]	[0.88–23.50]	[0.11–21.12]	[2.01–15.13]	[4.33–77.72]	–
Elementary school	1.23 (10/810)	1.56 (13/833)	3.57 (3/84)	6.06 (6/99)	1.61 (3/186)	0 (0/58)	2 (1/50)
	[0.47–2.00]	[0.72–2.40]	[0.74–10.08]	[1.36–10.76]	[0.33–4.64]	–	[0.05–10.65]
Middle school	0.13 (1/797)	1.33 (7/525)	0.87 (1/115)	0 (0/70)	0 (0/37)	1.85 (1/54)	0 (0/35)
	[0.003–0.70]	[0.35–2.31]	[0.02–4.75]	–	–	[0.05–9.89]	–
High school or higher	2.94 (1/34)	0 (0/37)	0 (0/1)	0 (0/16)	0 (0/10)	0 (0/3)	0 (0/3)
	[0.07–15.33]	–	–	–	–	–	–
*χ*^2^-value	61.825	19.603	3.934	5.278	10.248	25.262	1.271
*P*-value	< 0.0001	< 0.0001	0.269	0.153	0.017	< 0.0001	0.736

CI: confidence interval.

## Discussion

CE is the most prevalent form of echinococcosis in humans, and causes serious health impairments and significant economic burden globally. There have been a wealth of studies and reviews on echinococcosis in all aspects. Collective data indicated that at least 270 million people are at risk of CE in Central Asia, with the highest prevalence reaching 10% (range from 0.8 to 11.9%) in some Tibetan communities in western China. AE is also endemic in Central Asia and is recognized as a major problem in some Tibetan communities. The disease occurred in at least 21 of China’s 31 provinces/autonomous regions/municipalities, among them Guansu is one of the highly endemic areas. Tibet had the highest annual incidence of human CE (32 cases/100,000 inhabitants), followed by Qinghai (10.1/100,000), Ningxia (4.5/100,000), Xinjiang (3.0/100,000) and Gansu (0.9/100,000)^18,23^. A national epidemiological survey for major parasitic diseases in 2004 showed that the prevalence of CE in China was 1.08%^24^. The result of the present cross-sectional survey in 12 villages in Xiaonangou township of east Gansu showed a CE prevalence of 2.21%, corroborating the transmission remained high in this area. It was reported that the hepatic CE prevalence by US among Tibetans in mid-Gansu was found of 1.0% and 0.9%^25,26^ and 1.0% prevalence in Tibetan community found in south Gansu^23^. The CE prevalence varies in different parts of Gansu, depending on the environment parameters, residents’ life styles, the awareness on echinococcosis prevention and the local control strength. The reported AE cases in Gansu province were concentrated in south Gansu. A large focus of human AE was confirmed by mass US screening in the survey during 1994–1997 in south Gansu, showing a 3.4% prevalence^10^. Furthermore, liver US scan identified nine AE and one CE from 362 participants in west Gansu, demonstrating a AE and CE co-epidemic area^13^. The majority of residents in Gansu province engage in farming-pastoral activity, raising livestock including sheep and cattle, and guard dogs. Moreover, residents and animals usually drink the surface water collected through traditional cellar prone to contamination by fecal eggs excreted from infected dogs, creating potential transmission risks of echinococcosis^27^ (Fig. 2). In AE endemic areas, however, the land-use may change the landscape characteristics of scrub/grassland proportion. It was suggested that transmission of *E. multilocularis* in south Gansu may be related to a deforestation process driven by agriculture, probably resulting in the creation of optimal peri-domestic habitats for rodents that serve as intermediate host and subsequent development of a peri-domestic cycle involving dogs. However, deforestation practice dose not occur in east Gansu.

The analysis of the questionnaire responses indicates that the risks of echinococcosis transmission were highly related to gender, age and education background. The prevalence distribution by gender revealed that the prevalence in females was significantly higher than that in males, which conformed with the findings in other endemic areas^25,28,29^. In daily life, women perform most of the housework, taking care of livestock and dogs, and preparing dung fuel with bare hands. The dung may be contaminated with canine feces. They spent most of their time around tents and houses, where dogs and/or dog feces were present. Similarly, higher risk among females was also reported in Tibetan communities of Sichuan and Gansu province^10,30^. The age distribution in this survey demonstrated that the prevalence increased with age. Similar findings were reported from previous surveys in Tibetan communities and Qinghai province, indicating the age appeared to be a potential risk factor^28,30^. It may be attributed to the fact that the disease develops as a slow-growing mass in the body, with an incubation period for months or years; it requires a certain time of period for hydatid cyst or lesion to establish before being detectable by ultrasonography.

The prevalence surveillance in 2008–2014 in Yanghutaozi village indicates that the CE prevalence remained at about 1–3%, with the highest 3.35% in 2011, and the lowest 0.88% in 2014. Despite some control efforts were made in this area, the disease surveillance data did not show significant changes in prevalence. Nevertheless, our result on the other hand demonstrates that the transmission remained persistent during the years surveyed in this area. It was found that the prevalence surveillance showed an increasing trend with ages, which was also observed in the cross-sectional survey. In addition, the prevalence distribution by education level demonstrated again that the highest prevalence was seen in illiterate residents due to lack of disease knowledge and hygienic practice, thus at higher risk.

In the current survey, application of ultrasonography only on abdominal scanning may under-estimate the true prevalence of echinococcosis, due to the probability that the examinees, particularly among children, with lesions in other organs would possibly be missed, as it was reported that CE may affect multiple organs in younger population^31^. In addition, since the disease surveillance work did not include serology to confirm the suspected images; thus, the approach devoid of serology may influence the accuracy of prevalence estimation. For future study, we expect to include the serology in disease surveillance study, and applying bigger and similar sample size for screening each year.

In summary, the current study aims to understand the echinococcosis states in east Gansu province. The cross-sectional survey in 2011 found a prevalence of 2.21%, and the prevalence surveillance by ultrasonography in 2008–2014 revealed the prevalence ranging in 2–3%, with the highest 3.35% in 2011, and the lowest 0.88% in 2014, demonstrating the transmission remained high in east Gansu, in addition to south, west and mid-Gansu; Risk factors are found to be in association with the age, gender and education background. Higher prevalence was seen in females, elders (age > 60 years), and illiterates (*P* < 0.05).

## Data Availability

Data supporting the conclusions of this article are included in the article. The datasets used and/or analyzed during the current study are available from the corresponding author upon reasonable request.
